# Navigiert oder konventionell in der Acetabulumchirurgie: Vergleich der Positionsgenauigkeit am Beispiel der infraazetabulären Schraube

**DOI:** 10.1007/s00113-023-01304-1

**Published:** 2023-03-16

**Authors:** Stefano Pagano, Karolina Müller, Volker Alt, Günther Maderbacher, Dominik E. Holzapfel, Florian Baumann, Viola Freigang

**Affiliations:** 1 Klinik und Poliklinik für Unfallchirurgie, Universitätsklinikum Regensburg, Franz-Josef-Strauß-Allee 11, 93053 Regensburg, Deutschland; 2 Orthopädische Klinik für die Universität Regensburg, Asklepios Klinikum Bad Abbach, Kaiser-Karl V.-Allee 3, 93077 Bad Abbach, Deutschland

**Keywords:** Acetabulumchirurgie, Infraazetabuläre Schraube, Navigationssystem, Acetabulumfraktur, Vorderer und hinterer Pfeiler, Beckenchirurgie, Acetabular surgery, Infra-acetabular screws, Navigation system, Acetabular fracture, Anterior and posterior column, Pelvic surgery

## Abstract

**Hintergrund:**

Behandlungsprinzip der gelenkerhaltenden Therapie von Acetabulumfrakturen ist die anatomische Reposition der gelenktragenden Elemente und die interne Osteosynthese. Um den vorderen und den hinteren Pfeiler gegeneinander zu stabilisieren, wird die infraazetabuläre Schraube (IAS) im klinischen Alltag regelhaft eingesetzt.

**Ziel:**

Ziel der vorliegenden Studie ist es, die Lage der IAS im infraazetabulären Korridor nach navigierter Platzierung mit der nach Freihandplatzierung zu vergleichen.

**Material und Methode:**

Die Lage der Schraube wurde bei 42 Patienten mithilfe multiplanarer Rekonstruktionen evaluiert. Bei 30 Patienten wurde diese freihandplatziert, bei 12 Patienten mittels bildgestützter Navigation. Neben der Vermessung der Schraubenlage wurden demografische Daten, Operationszeit, Strahlenbelastung sowie Blutverlust erhoben.

**Ergebnisse:**

Der überwiegende Teil der Patienten war männlich (86 %), das mediane Alter lag bei 67 Jahren und der mediane BMI bei 25 kg/m^2^. Die mediane Operationszeit betrug 166 min, und die mediane Blutverlustmenge lag bei 900 ml. Die adjustierten Werte in der gesamten Stichprobe bezüglich der Position der Schrauben lagen bei: Abstand Schraube zum Knorpel Mittelwert (MW) = 3,8 mm, Abstand Schraube zum Korridorzentrum MW = 3,5 mm, Winkel Schraube zum Korridor MW = 1,4°. Die zwei Gruppen unterschieden sich nicht in den demografischen Parametern sowie in der Genauigkeit der Positionierung der Schrauben (*p*-Werte > 0,05). In der navigierten Gruppe zeigten sich eine längere Strahlungszeit und höhere Strahlendosis im Vergleich zur Gruppe ohne Navigation (*p*-Werte < 0,001).

**Schlussfolgerung:**

Die beiden Verfahren sind bei entsprechender Erfahrung hinsichtlich der Genauigkeit vergleichbar. Hinsichtlich weiterer perioperativer Parameter wie Strahlenbelastung und geplanter Operationsdauer sollten auch patientenbezogene Faktoren berücksichtigt werden.

## Abstract (en)

**Background:**

The principle of joint-preserving treatment of acetabular fractures is the anatomic reduction of joint-bearing elements and internal osteosynthesis. For stabilization of the anterior and posterior columns against each other, the infra-acetabular screw (IAS) is regularly used in the clinical routine.

**Objective:**

The aim of this study was to compare the position of the IAS in the infra-acetabular corridor after navigated placement and after freehand placement.

**Material and method:**

The position of the screw was evaluated in 42 patients using multiplanar reconstructions. The screw was placed freehand in 30 patients and using image-guided navigation in 12 patients. In addition to measurement of the position of the screw, demographic data, operating time, radiation exposure and blood loss were recorded.

**Results:**

The vast majority of the patients were male (86%), the median age was 67 years and the median body mass index (BMI) was 25 kg/m^2^. The median operating time was 166 min and the median blood loss was 900 ml. The adjusted values in the whole sample considering the position of the screw were: distance of screw to cartilage mean value (m) = 3.8 mm, distance of screw to corridor center m = 3.5 mm and angle of screw to corridor m = 1.4°. There were no differences between the groups in the demographic parameters and the accuracy of positioning of the screw (*p*-value > 0.05). There was a longer irradiation time and a higher radiation dose in the navigated group compared to the group without navigation (*p*-value < 0.001).

**Conclusion:**

With appropriate experience both procedures are comparable taking into account the accuracy. Taking into consideration other perioperative parameters, such as radiation exposure and planned operating time, patient-related factors should be taken into consideration.

**Graphic abstract:**

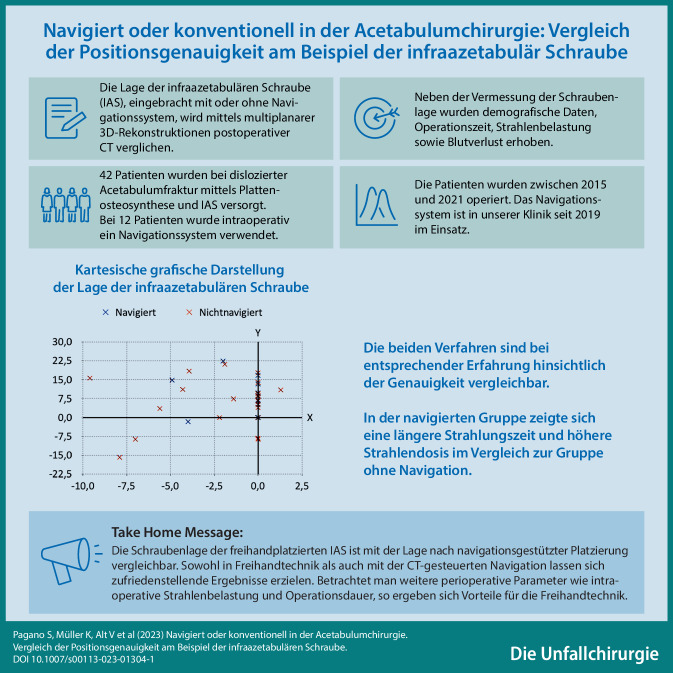

## Einleitung

Der Schlüssel zur erfolgreichen operativen Behandlung von Acetabulumfrakturen ist neben der exakten anatomischen Reposition die stabile Fixation, um eine sekundäre Dislokation und dadurch eine posttraumatische Koxarthrose zu vermeiden [1–3] Durch die demografische Entwicklung der Gesellschaft treten zunehmend mehr Altersfrakturen mit Beteiligung des vorderen Pfeilers auf. Diese Entwicklung bewirkt, dass bei reduzierter Knochenqualität eine höhere Primärstabilität der Osteosynthese erforderlich ist [4].

Frakturformen mit Dissoziation von vorderem und hinterem Pfeiler und Dislokation der quadrilateralen Fläche haben ein hohes Potenzial für einen sekundären Repositionsverlust und die Entstehung von zentraler Luxation [5]. Somit ist es notwendig, den anterioren an den posterioren Pfeiler stabil zu fixieren, um den von Letournel postulierten periazetabulären Rahmen wiederherzustellen. Die von Culemann et al. beschriebene infraazetabuläre Schraube (IAS) wird ca. 1 cm kaudal und medial der Eminentia iliopectinea (IPE) und parallel zur quadrilateralen Fläche eingebracht [9]. Der infraazetabuläre Korridor (IAC) entspricht der Köhler-Tränenfigur im a.-p.-Röntgenbild des Beckens. Der IAC hat einen Durchmesser von wenigen Millimetern, was bei Fehlpositionierung zur Perforation nach intrapelvin oder in das Hüftgelenk führen kann [6].

Die zunehmenden Entwicklungen im Bereich bildgestützten Navigation bieten eine Vielzahl an Einsatzmöglichkeiten in der operativen Versorgung von komplexen Frakturen [7–9].

Inwieweit die chirurgische Navigation Komplikationen reduzieren kann und der Freihandplatzierung anhand anatomischer Landmarken überlegen ist, ist bisher wenig untersucht und soll Gegenstand dieser Studie sein.

## Methodik

Die vorliegende Studie vergleicht die Lage der IAS, eingebracht mit oder ohne Navigationssystem bei 42 Patienten, die zwischen 2015 und 2021 bei dislozierter Fraktur des Acetabulums mithilfe einer Plattenosteosynthese versorgt wurden.

Als Einschlusskriterien wurden definiert:

- dislozierte Fraktur des Acetabulums,
- operative Frakturversorgung,
- Stabilisierung mittels IAS.

Ausschlusskriterien waren:

- inkomplette Bildgebung,
- beidseitige Versorgung,
- konservative Therapie oder primäre Endoprothesenversorgung.

**Figure 1:**
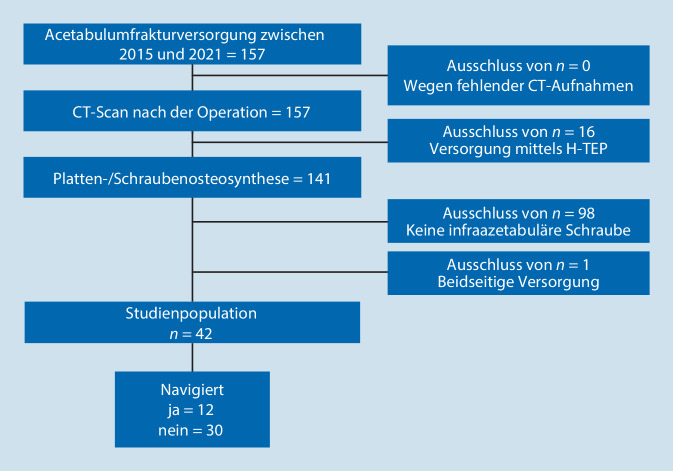

Innerhalb der letzten 6 Jahre wurden in der Abteilung der Unfallchirurgie des Universitätsklinikums Regensburg 157 Patienten mit einer Acetabulumfraktur operativ versorgt. Alle diese Patienten erhielten prä- und postoperativ CT-Aufnahmen. Es wurden 16 Patienten von der Studie ausgeschlossen, da sie eine primäre Hüftendoprothesenversorgung benötigten. Von den verbliebenen 141 Patienten erhielten 98 keine IAS, und ein Patient wurde bei beidseitiger Frakturversorgung ausgeschlossen. Somit ergab sich eine Studienpopulation von 42 Patienten. In beiden Gruppen wurden die Operationen durch die gleichen Operateure durchgeführt. Im Rahmen der Behandlung erhielten 12 Patienten eine navigationsgestützte Operation, während bei 30 Patienten die konventionelle Freihandtechnik und zweidimensionale Fluoroskopie zur osteosynthetischen Versorgung angewendet wurde (Abb. 1).

**Figure 2:**
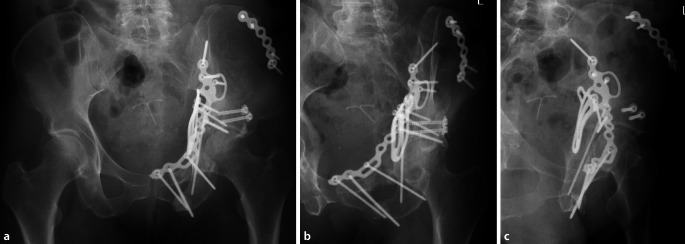

Die meisten Patienten wurden über einen modifizierten Stoppa-Zugang operiert. In 3 Fällen in der nichtnavigierten Gruppe war ein ilioinguinaler Zugang notwendig. Nach offener Reposition der Acetabulumfraktur wurde die Reposition mittels Fluoroskopie in a.-p.- und in Ala‑/Obturator-Aufnahmen kontrolliert (Abb. 2). In der navigierten Gruppe erfolgte zusätzlich eine 3D-Fluoroskopie mit digitaler Volumentomographie (Ziehm RFD 3D, Fa. Ziehm Imaging, Nürnberg, Deutschland) als Datengrundlage für die Navigation. Für die präoperative Planung und Navigation wurde das System der Fa. Brainlab (BrainLab curve, Fa. Brainlab, München, Deutschland) verwendet. Mit der chirurgischen Navigation kann der Bohrvorgang auf Basis der intraoperativen Bildgebung in Echtzeit kontrolliert werden. Über das optische Tracking des Bohrers verändert sich die multiplanare Reformation (ähnlich einer CT-Rekonstruktion) dynamisch, sodass der Bohrkanal präzise dargestellt wird. Das Eindrehen der Schraube erfolgt ohne zusätzliche Bildsteuerung. Der idealer Eintrittspunkt der IAS wurde von Culemann et al. beschrieben [9, 10]. Die übrigen Operationsschritte zur Frakturstabilisierung wurden in der Technik nach Letournel durchgeführt [11].

**Figure 3:**
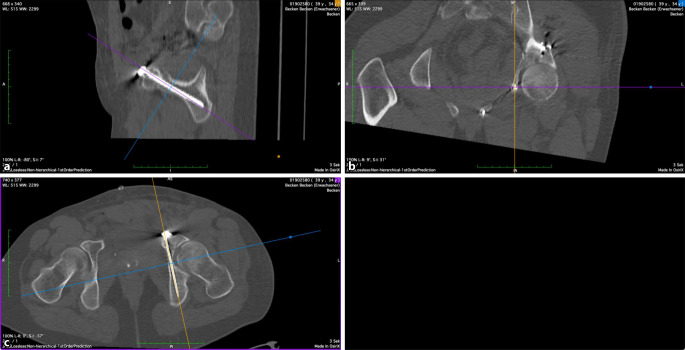

**Figure 4:**
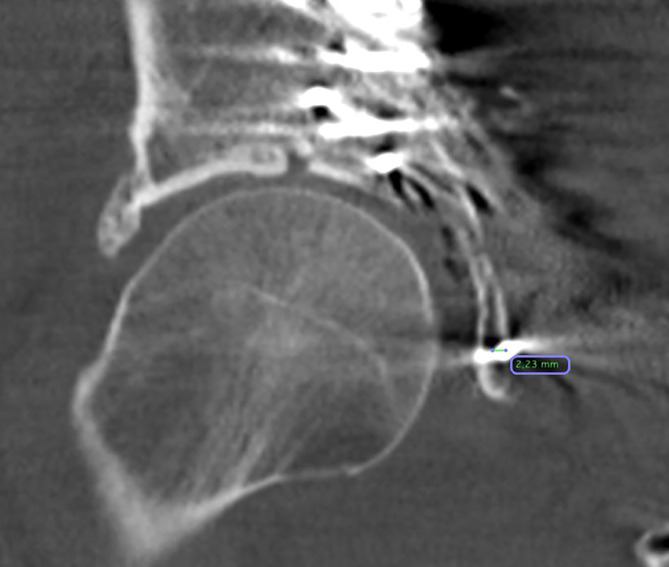

**Figure 5:**
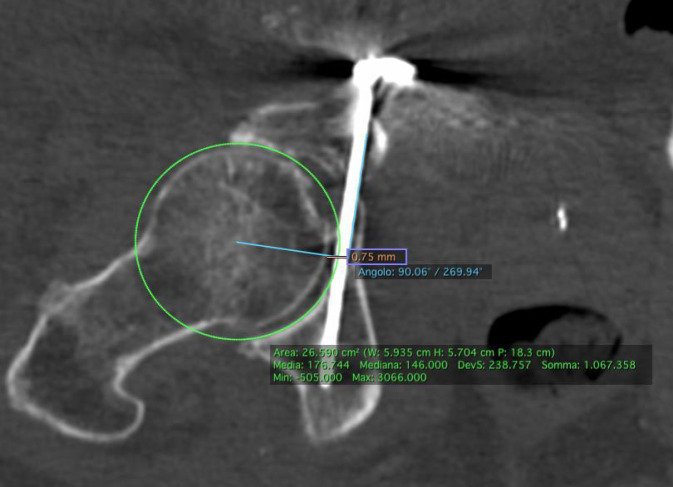

**Figure 6:**
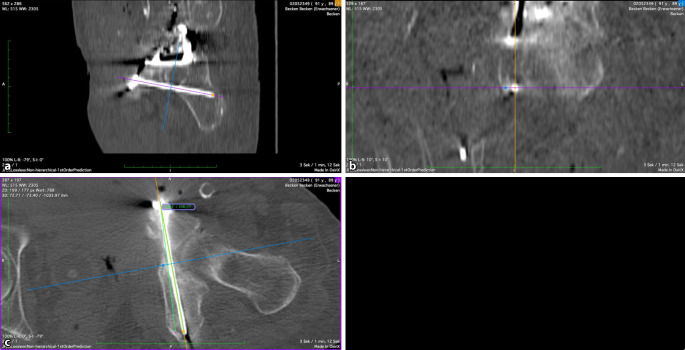

Die Analyse der Schraubenlage wurde mit der Open-Source-Software OsiriX Lite (Fa. Pixmeo, Bernex, Schweiz) durchgeführt. Die postoperativen Computertomographie(CT)-Scans aller Patienten wurden multiplanar 3D-rekonstruiert. Der IAC wurde nach der von Egli und Kanezaki beschriebenen Technik identifiziert [12, 13]. Der infraazetabuläre Diameter (IAD) wurde als die schmalste Stelle des Korridors auf der Inlet-Aufnahme des Beckens in kaudaler Inklination definiert (Abb. 3). Die Schrauben-Korridor-Distanz (SCD) wurde auf der axialen Aufnahme als kürzeste Distanz zwischen dem Zentrum des IAC und dem Zentrum der Schraube festgelegt (Abb. 4). Der Schrauben-Knorpel-Abstand (SFCD) wurde nach Darstellung der gesamten Schraubenlänge im CT als Abstand des Schraubenkopfes zum Femurkopfknorpel definiert (Abb. 5). Der Schrauben-Korridor-Winkel (SCA) wurde definiert als der Winkel zwischen der Achse, die durch das Zentrum der Schraube verläuft, und der Achse, die durch das Zentrum des IAC verläuft. Beide Achsen haben ihren Ursprung an der Eintrittsstelle der Schraube in den Knochen (Abb. 6).

Die vorliegende Studie wurde durch die Ethikkommission der Universität Regensburg genehmigt (Review Board Nummer: 21-2235-104). Die Studie wurde in Übereinstimmung mit der Deklaration von Helsinki von 1964 durchgeführt.

Die statistische Analyse erfolgte mittels der Software SPSS (Version 28, SPSS Inc, Chicago, IL, USA). Das Signifikanzlevel wurde auf *p*_zweiseitig_ ≤ 0,05 für alle Tests festgelegt. Es fand keine Adjustierung für multiples Testen statt. Die deskriptiven Daten werden als absolute (*n*) und prozentuale Häufigkeiten (%) für kategoriale Variablen, Mittelwert (MW) und Standardabweichung (SD) sowie Median und Interquartil-Range (IQR) für stetige Variablen angegeben. Je nach Skalenniveau und Verteilung der stetigen Variablen wurden Exakte Tests nach Fisher, *t*-Tests für unabhängige Stichproben und U‑Tests für den Vergleich der demografischen und klinischen Parameter zwischen Patienten mit Navigation vs. ohne Navigation verwendet. Zusätzlich wurden Kovarianzanalysen (ANCOVA) verwendet, um beide Gruppen im SCD, SFCD und SCA adjustiert für die Co-Faktoren Schraubenlänge und IAD zu vergleichen.

**Figure 7:**
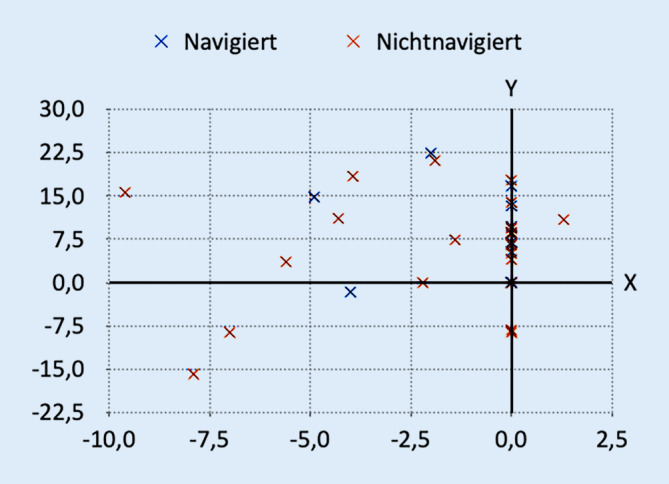

Um auf einen Blick den Unterschied in der Positionierung der Schraube in den beiden Gruppen zu erfassen, wurde ein kartesisches Diagramm erstellt (Abb. 7).

**Figure 8:**
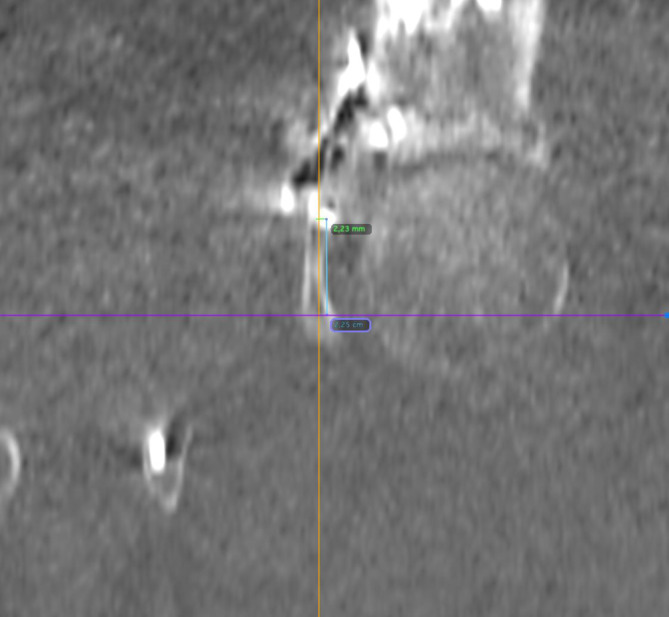

Die Koordinaten wurden bestimmt, indem für jeden Patienten der Abstand zwischen der Schraube und den beiden Achsen eines Diagramms gemessen wurde, dessen Ursprung in dem Zentrum des IAC liegt (Abb. 8).

## Ergebnisse

### Stichprobebeschreibung

In dieser Studie wurden 36 Männer und 6 Frauen eingeschlossen. Das mediane Alter der gesamten Studienpopulation lag bei 67. Der mediane BMI war 24,8 kg/m^2^.

In der Studienpopulation hatten 17 Patienten eine Fraktur des vorderen Pfeilers mit hinterer Hemiquerfraktur, zwei Patienten eine T‑Fraktur, 16 Patienten eine Zwei-Pfeiler-Fraktur, 5 Patienten eine Fraktur des vorderen Pfeilers, ein Patient eine Querfraktur und ein Patient eine Vorderwandfraktur. 29 Patienten (69 %) erlitten ein isoliertes Beckentrauma, während 13 Patienten (31 %) Begleitverletzung aufwiesen.

Alle Patienten in der navigierten Gruppe wurden über einen modifizierten Stoppa-Zugang versorgt. Bei 6 Patienten (50 %) war zusätzlich die Eröffnung des ersten Fensters des ilioinguinalen Zugangs notwendig.

In der nichtnavigierten Gruppe wurde ein modifizierter Stoppa-Zugang bei 27 Patienten angewendet (90 %); in 8 Fällen wurde der Zugang durch das erste Fenster ergänzt (30 %). Bei der übrigen 3 Patienten wurde ein ilioinguinaler Zugang verwendet.

Die mediane Operationszeit lag in der CT-gesteuert navigierten Gruppe bei 187 min und in der nichtnavigierten, freihandplatzierten Gruppe bei 152 min. Es zeigte sich ein medianer Blutverlust von 900 ml der navigierten Gruppe und von 775 ml in der nichtnavigierten Gruppe. Diese Unterschiede zeigten sich nicht statistisch signifikant.

Es zeigte sich ein medianer Blutverlust von 900 ml (IQR = 550–1045), wenn der Stoppa-Zugang allein benutzt wurde. Bei der zusätzlichen Anwendung des ersten Fensters zeigte sich ein medianer Blutverlust von 750 ml (IQR = 495–2200) (*p* = 0,936).

Die mediane Strahlungsdosis und Strahlungszeit war zwischen den Gruppen signifikant unterschiedlich und betrug in der navigierten Gruppe 1272,8 cGycm^2^ und 115,0 s, in der nichtnavigierten Gruppe 223,9 cGycm^2^ und 37 s (*p*-Werte < 0,001) (Tab. 1).

**Table 1:** 

	Gesamt *n* = 42		Navigiert *n* = 12		Nichtnavigiert *n* = 30		*p*-Wert
Alter (Median, IQR)	67	(57–79)	65	(45–82)	67	(59–78)	0,791
BMI (Median, IQR)	24,8	(21,8–28,2)	23,6	(21,4–29,9)	25,2	(22,5–28,2)	0,578
Operationszeit in Minuten (Median, IQR)	166	(133–220)	187	(156–233)	152	(127–220)	0,064
Röntgendosis (Median, IQR)	443	(132–1170)	1273	(596–1935)	224	(101–607)	< 0,001
Röntgendauer in Sekunden (Median, IQR)	67	(20–124)	115	(88–206)	37	(15–81)	< 0,001
Blutverlust (Median, IQR)	900	(500–1200)	900	(547–1000)	775	(500–1350)	0,655
Korridordiameter (MW ±SD)	4,7	(±1,1)	4,7	(±1,0)	4,6	(±1,2)	0,919
Schraube-Knorpel-Abstand (SFHD) (MW, 95 %-KI)	3,8	(2,7–4,8)	3,3	(1,6–5,0)	4,2	(3,1–5,3)	0,371
Schraube-Korridor-Abstand (SCD) (MW, 95 %-KI)	3,5	(2,3–4,6)	3,6	(1,7–5,5)	3,3	(2,1–4,5)	0,815
Schraube-Korridor-Winkel (SCA) (MW, 95 %-KI)	1,4	(0,8–2,1)	1,1	(0,1–2,2)	1,7	(1,0–2,4)	0,392
Schraubenlänge (Median, IQR)	95	(90–100)	95	(90–100)	95	(90–100)	0,831
*Geschlecht (n, %)*							0,655
Männlich	36	(85,7 %)	11	(91,7 %)	25	(83,3 %)	
Weiblich	6	(14,3 %)	1	(8,3 %)	5	(16,7 %)	
*Begleitverletzung*							0,463
Ja	13	(31 %)	5	(41,7 %)	8	(26,7 %)	
Nein	29	(69 %)	7	(58,3 %)	22	(73,3 %)	
*Frakturtyp*							–
T‑Fraktur	2	(4,8 %)	1	(8,3 %)	1	(3,3 %)	
Zweipfeiler Fraktur	16	(38,1 %)	5	(41,7 %)	11	(36,7 %)	
Vorderer Pfeiler mit hinterer Hemiquerfraktur	17	(40,5 %)	5	(41,7 %)	12	(40,0 %)	
Vorderer Pfeiler	5	(11,9 %)	1	(8,3 %)	4	(13,3 %)	
Querfraktur	1	(2,4 %)	0	(0,0 %)	1	(3,3 %)	
Vorderwandfraktur	1	(2,4 %)	0	(0,0 %)	1	(3,3 %)	
*Zugang*							–
Modifizierter Stoppa-Zugang	*25*	*(59.5* *%)*	*6*	*(50* *%)*	*19*	*(63.3* *%)*	
Stoppa-Zugang mit Eröffnung des ersten Fensters	*14*	*(33,3* *%)*	*6*	*(50* *%)*	*8*	*(26,7* *%)*	
Ilioinguinaler Zugang	*3*	*(7,2* *%)*	*–*	*0*	*3*	*(10* *%)*	

*IQR* Interquartil-Range, *MW* Mittelwert, *SD* Standardabweichung, *KI* Konfidenzintervall, *SFHD* „screw-femoral-head distance“, *SCD* „screw-corridor distance“, *SCA* „screw-corridor-angle“

### Schraubenlageanalyse

Der SCD, SFCD sowie SCA unterscheiden sich nicht signifikant in den beiden Gruppen (*p*-Wert > 0,050, adjustiert für Schraubenlänge und IAD).

Die mediane Länge der Schraube war in beiden Gruppen bei 95 mm ± 5 mm (*p* = 0,831).

**Figure 9:**
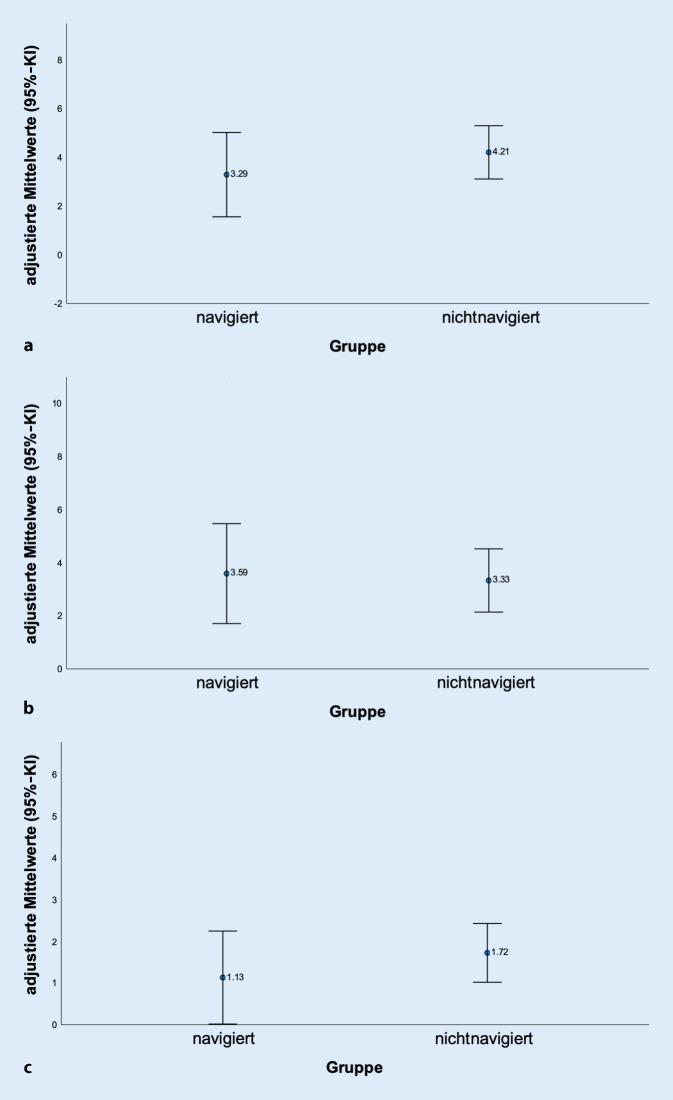

Bei etwa gleichem Korridordiameter von 4,7 ± 1,0 mm in der navigierten Gruppe und 4,6 ± 1,2 mm in der freihandplatzierten Gruppe zeigte sich der SFHD mit 3,3 mm in der navigierten Gruppe und mit 4,2 mm in der freihandplatzierten Gruppe (*p* = 0,371). Der SCD betrug 3,6 mm in der navigierten Gruppe und 3,3 mm in der nichtnavigierten Gruppe (*p* = 0,815). Der SCA betrug in der navigierten Gruppe 1,2° und 1,7° in der nichtnavigiert platzierten Gruppe (*p* = 0,392) (Abb. 9).

## Diskussion

Die operative Versorgung von Acetabulumfrakturen ist anspruchsvoll. Die anatomische Rekonstruktion der Gelenkfläche und eine stabile Osteosynthese sind die Grundpfeiler der gelenkerhaltenden Chirurgie. Gelingt es nicht, die Anatomie zu rekonstruieren oder tritt eine sekundäre Dislokation aufgrund ungenügender Primärstabilität auf, kommt es zudem zu einer Mehrbelastung des Acetabulumdoms, was über eine entstehende „zentralen Subluxation“ zu rascher Destruktion des Gelenkes führen kann [14].

Die Überalterung der Gesellschaft führt in Deutschland zu einer Zunahme der Altersfrakturen des Acetabulums [15]. Diese sind durch eine deutlich schlechtere Knochenqualität und ein typisches Frakturmuster mit Beteiligung des vorderen Pfeilers charakterisiert [16]. Daten des Statistischen Bundesamtes zeigen, dass im Jahr 2019 über 70 % der Patienten mit einer Acetabulumfraktur über 70 Jahre alt waren [17]. Auch in dieser Studie zeigte sich ein durchschnittliches Alter von 70 Jahren.

Die Navigation ist in der Abteilung der Unfallchirurgie des Universitätsklinikums Regensburg seit 2019 im Einsatz. Seitdem wird die Mehrzahl der Patienten mit Acetabulumfraktur navigationsgestützt operiert. Dementsprechend sind die Patienten aus der nichtnavigierten Kontrollgruppe in der Zeit vor der Anschaffung der CT-Navigation operiert worden. Der Altersunterschied zwischen den beiden Gruppen zeigt somit auch die Entwicklung der letzten Jahre. Zwar ist eine Fraktur des Acetabulums eine seltene Verletzung, aktuelle Daten von Rupp et al. zeigen aber, dass keine Fraktur in den vergangenen 10 Jahren so stark zugenommen hat wie die des Acetabulums [17].

Die Zunahme der Altersfrakturen hat auch zu einer Entwicklung der ventralen Zugangswege und neuer Operationstechniken geführt. Der intrapelvine oder Pararectus-Zugang bietet deutlich mehr Übersicht als der klassische ilioinguinale Zugang [18]. Anatomische Plattensysteme mit Abstützung der quadrilateralen Fläche bieten eine höhere Primärstabilität – ein Vorteil bei Altersfrakturen mit reduzierter Knochenqualität [19]. Zur Verbesserung der Stabilität hat sich sowohl in biomechanischen Studien als auch in der klinischen Beobachtung das zusätzliche Einbringen einer IAS bewährt. Letournel beschrieb 1993 erstmals eine periazetabuläre Rahmenfixation für besondere Frakturkonstellationen [20].

Der Korridor für das Einbringen der IAS projiziert sich im Beckenübersichtsröntgen auf die Köhler-Tränenfigur und liegt damit in einer „unsicheren Zone“ für die Platzierung von Schrauben in der Umgebung des Acetabulums [21].

Die konventionelle Platzierung der IAS in Freihandtechnik birgt das Risiko einer Schraubenabweichung nach intraartikulär oder intrapelvin. Während die intrapelvine Perforation in der Regel als unkritisch gesehen wird, stellt die intraartikuläre Schraubenlage einen Revisionsgrund dar [22].

Um die optimale Positionierung zu erreichen, ist die anatomische Reposition des vorderen gegen den hinteren Pfeiler essenziell. Bei nichtanatomischer Reposition der Pfeiler ist u. U. die Integrität des IAC nicht gegeben, weshalb die Platzierung einer IAS kritisch zu prüfen ist. Eine intraoperative 3D-Bildgebung nach der Reposition ist hilfreich, um den Knochenkanal zu beurteilen. Für die Osteosynthese des Acetabulum werden in der Regel 3,5-mm-Schrauben verwendet, auch weil der Korridor für die IAS keine großen Schraubendurchmesser zulässt. Anders also als bei der hinteren Pfeilerschraube oder der Kriechaschraube des Schambeinastes, bei der der Korridor auch größere Diameter zulässt.

Diese Bildgebung kann auch als Grundlage für die Navigation genutzt werden. Wie die vorgelegte Studie zeigt, geht die intraoperative Schnittbilddiagnostik aber auch mit einer deutlichen Zunahme der Strahlenexposition von 224 cGycm^2^ auf 1273 cGycm^2^ einher. Dies ist jedoch auch der Methodik geschuldet, da nur in der Navigationsgruppe eine intraoperative 3D-Bildgebung erfolgte. Die Unterschiede in der Strahlenbelastung wären weniger groß, wenn man auch bei den nichtnavigierten Fällen intraoperativ eine CT-Kontrolle der Reposition und Implantatlage durchgeführt hätte.

Der Einsatz der Navigation führte in der vorliegenden Studie auch zu einer Verlängerung der Operationsdauer um durchschnittlich 35 min. Die Erstellung des Bilddatensatzes, inclusive zusätzlicher Abdeckung des Operationsgebietes, spielt dabei ebenso eine Rolle wie das Anbringen der optischen Referenzkugeln an der Crista iliaca. Das zusätzliche Trauma durch die perkutane Platzierung von 2 Schanz-Schrauben für die Referenz muss ebenso in Betracht gezogen werden. In diesem Studienkollektiv musste zudem in der navigierten Gruppe das erste Fenster häufiger auch eröffnet werden.

Neben der höheren Präzision bietet die chirurgische Navigation auch die Möglichkeit, Eingriffe minimal-invasiv durchzuführen. Zwar gab es in der vorgelegten Studie in beiden Gruppen Patienten, bei denen im postoperativen Computertomogramm eine intraartikuläre Schraubenlage festgestellt wurde, da die Fossa acetabuli jedoch ausreichend tief war, kam es in keinem Fall zu einem Konflikt zwischen der Schraube und dem Femurkopf, weshalb keine Revision nötig wurde. Nur eine Affektion des femoralen Kontaktknorpels wird als Revisionsgrund gesehen, weshalb auch hier die Vermessung anhand des Femurkopfknorpels erfolgte.

Die geringe Inzidenz von Acetabulumfrakturen führt dazu, dass diese Patienten meist in dafür spezialisierten Zentren versorgt werden. Die Tatsache, dass im dargestellten Patientengut keine revisionspflichtige Lageabweichung einer IAS beobachtet wurde, bedeutet nicht, dass die Platzierung dieser Schraube als unkritisch gelten kann. Der Einstieg in die Acetabulumchirurgie ist von einer flachen Lernkurve gekennzeichnet, und die konventionelle Schraubenplatzierung im IAC bleibt eine anspruchsvolle Prozedur [23]. Umso wichtiger ist es, mögliche Vorteile durch Innovationen wie die chirurgische Navigation im klinischen Einsatz wissenschaftlich zu untersuchen.

## Fazit für die Praxis

Die Schraubenlage der Freihand platzierten IAS ist mit der Lage nach navigationsgestützter Platzierung vergleichbar. Sowohl in Freihandtechnik als auch mit der CT-gesteuerten Navigation lassen sich zufriedenstellende Ergebnisse erzielen. Betrachtet man weitere perioperative Parameter wie intraoperative Strahlenbelastung und Operationsdauer, so ergeben sich Vorteile für die Freihandtechnik.
